# Imbalance in the Expression of Genes Associated with Purinergic Signalling in the Lung and Systemic Arteries of COPD Patients

**DOI:** 10.1038/s41598-019-39233-y

**Published:** 2019-02-26

**Authors:** Oriol Careta, Ester Cuevas, Mariana Muñoz-Esquerre, Marta López-Sánchez, Yuliana Pascual-González, Jordi Dorca, Elisabet Aliagas, Salud Santos

**Affiliations:** 1 Pneumology Research Group, Institut d’Investigació Biomèdica de Bellvitge – IDIBELL, L’Hospitalet de Llobregat, Barcelona, Spain; 2 Department of Respiratory Medicine, Bellvitge University Hospital, L’Hospitalet de Llobregat, Barcelona, Spain; 3 Department of Clinical Sciences, University of Barcelona, L’Hospitalet de Llobregat, Barcelona, Spain; 4 Research Network in Respiratory Diseases (CIBERES), Madrid, Spain

## Abstract

Growing evidence indicates that purinergic signalling is involved in the pathogenesis of chronic obstructive pulmonary disease (COPD) and in the vascular remodelling that occurs in other disorders; however, its role in initial vascular changes of COPD is not entirely known. We hypothesised that expression of genes regulating extracellular ATP and adenosine levels would be altered in the lung and systemic arteries of COPD patients. Quantitative real-time PCR was performed to analyse the relative expression of 17 genes associated with purinergic signalling and inflammation in lungs and intercostal arteries of never smokers (NS) (n = 16), non-obstructed smokers (NOS) (n = 17) and COPD patients (n = 21). Gene expression of ATP-degrading enzymes was decreased in both tissues of NOS and COPD patients compared to NS. *NT5E* expression (gene transcribing for an AMP hydrolyzing ectonucleotidase) was increased in both tissues in NOS compared to the other groups. P1 and P2 receptors did not show changes in expression. Expression of genes associated with inflammation (interleukin-13) was upregulated only in lung tissues of COPD. These findings suggest that the expression of different extracellular ATP-degrading enzymes is altered in smokers (NOS and COPD patients), promoting inflammation. However, the high *NT5E* expression found only in NOS could compensate this inflammatory environment.

## Introduction

Chronic obstructive pulmonary disease (COPD) is a highly prevalent chronic respiratory disease and a major cause of mortality and morbidity worldwide^1^. Smoking has long been recognised as the main risk factor^2^, but not all smokers develop the disease. The factors that determine the development of COPD in susceptible smokers are complex and may involve genetic and epigenetic factors, altered immune regulation, and abnormal repair mechanisms^3,4^.

COPD is associated with vascular alterations in both pulmonary and systemic arteries^5^. In advanced stages of the disease, the risk of developing pulmonary hypertension increases due to the loss of pulmonary vessels caused by emphysema, pulmonary vascular remodelling, and chronic hypoxaemia^6^. However, in the pulmonary arteries of patients with moderate COPD, an association between endothelial dysfunction^6^, structural changes^7^, and the presence of an inflammatory infiltrate in the adventitial layer^8^ has been also described.

Recently, morphometric studies carried out on the systemic and pulmonary arteries of patients with mild-moderate COPD have demonstrated a significant correlation between the initial changes that occur in the walls of both systemic and pulmonary arteries. These systemic and pulmonary changes are characterised by a thickening of the intima and a decrease in the vascular lumen, which are observed to a greater degree in COPD patients than in smokers without airflow obstruction^9^. To date, the mediators involved in these processes are not well known.

Numerous studies have indicated that purinergic signalling plays a major role in the development of COPD^10–12^ and in the vascular remodelling that occurs in other disorders like idiopathic pulmonary fibrosis^13^. Purinergic signalling involves purines (ATP and its hydrolysis products) and pyrimidines (mainly UTP) that act as extracellular ligands for the widely-expressed purinergic receptors, P2 (activated by nucleoside tri-/diphosphates among others) and P1 (activated by adenosine and others)^14^. It is well known that ATP and adenosine levels are increased in the lungs of COPD patients^15,16^, which could be associated with COPD development. Extracellular levels of ATP and adenosine are controlled by membrane proteins called ectonucleotidases, which include four families: the ectonucleoside triphosphate diphosphohydrolase (ENTPDase) family, the ectonucleotide pyrophosphatase/phosphodiesterase (ENPP) family, the alkaline phosphatase (AP) family, and the 5′-nucleotidase family, of which only one member (NT5E/CD73) is expressed on the membrane^17^.

The present study explored the expression of the genes involved in purinergic signalling and inflammation in the lung and vascular systemic tissues of never smokers (NS), non-obstructed smokers (NOS) and stable COPD patients. We performed quantitative real-time PCR on peripheral lung tissue and intercostal arteries (representative systemic vascular tissue) to examine the expression of 17 genes of interest.

## Results

### Gene expression studies in the lungs of NOS and COPD patients compared to NS

#### Gene expression of ATP-degrading enzymes and DPP4

**Figure 1:**
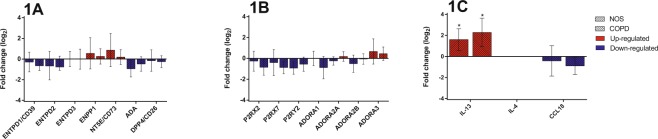
Gene expression analyses in the lung. Changes in the mRNA expression of genes for: NOS vs. NS and COPD vs. NS. Results are expressed as fold change (log_2_) relative to NS. Bar plots represent median ± log_2_ of RQmax and log_2_ of RQmin. **(A)** The expression of *ENTPD1*, *ENTPD2*, *ADA* and *DPP4* was downregulated in NOS and COPD patients. *ENPP1* expression was upregulated in NOS and COPD patients while *NT5E* expression was upregulated in NOS and unchanged in COPD. No changes were found in *ENTPD3* expression. **(B)** *P2RX2*, *P2RX7* and *P2RY2* expression was downregulated in COPD patients and NOS as well as the expression of *ADORA1* in COPD patients. *ADORA2A* and ADORA2B expression levels were similar to NS in both groups. *ADORA3* expression in NOS and COPD patients along with *ADORA2A* expression in COPD patients was upregulated. No changes were found in *ADORA1* expression in NOS. **(C)** *IL-13* expression was upregulated whereas *CCL18* expression was downregulated in NOS and COPD patients. No expression of *IL-4* was found. *Significantly different from NS (*p* < 0.05).

The gene expression of ATP-degrading enzymes and DPP4, which binds adenosine deaminase (ADA), is shown in Fig. 1A. All the ATP-degrading enzymes studied (*ENTPD1*, *ENTPD2* and *ENTPD3*) showed a similar expression pattern in both NOS and COPD patients, with decreases in *ENTPD1* and *ENTPD2* expression and no changes in *ENTPD3* expression. *ENPP1* expression was upregulated, while the gene expression of the adenosine-producing enzyme NT5E*/*CD73 was up to one-fold higher in NOS, but unchanged in COPD patients. *ADA* and *DPP4* expression was downregulated in both NOS and COPD patients.

#### Gene expression of P1 and P2 receptors

The gene expression of the purinergic P1 and P2 receptors is shown in Fig. 1B. In the lungs of NOS and COPD patients, gene expression of all the ATP receptors studied (*P2RX2*, *P2RX7* and *P2RY2*) was downregulated. In the case of adenosine receptors, *ADORA1* expression was downregulated only in COPD patients, while *ADORA3* expression was upregulated in both groups. *ADORA2A* and *ADORA2B* expression in NOS and COPD patients was similar to that in NS.

#### Expression of the genes associated with inflammation

Among the three genes encoding inflammatory molecules (*IL-13*, *IL-4* and *CCL18*) that were analysed in pulmonary tissue (Fig. 1C), only *IL-13* expression showed significant changes, as expected, with increases observed in both NOS and COPD patients. *IL-4* expression was not amplified probably due to the lack of tissue expression. *CCL18* expression was decreased in NOS and COPD patients compared to NS.

### Gene expression studies in the intercostal arteries of NOS and COPD patients compared to NS

#### Gene expression of ATP-degrading enzymes and DPP4

**Figure 2:**
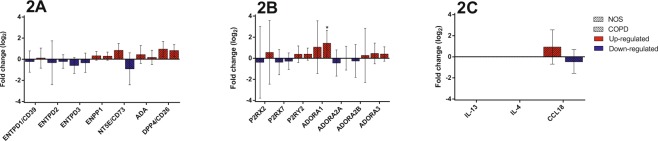
Gene expression studies in the intercostal arteries. Changes in the mRNA expression of genes for: NOS vs. NS and COPD vs. NS. Results are expressed as fold change (log_2_) relative to NS. Bar plots represent median ± log_2_ of RQmax and log_2_ of RQmin. **(A)** *ENTPD2* and *ENTPD3* expression was downregulated in NOS and COPD patients, as well as *ENTPD1* in NOS and *NT5E* in COPD patients. *ENPP1*, *ADA* and *DPP4* expression levels were increased in both groups. *ENTPD1* expression in COPD patients and *NT5E* expression in NOS were also upregulated. **(B)** *P2RX2*, *ADORA2A* and *ADORA2B* expression was downregulated in NOS. *P2RX7* expression was also downregulated in both groups. *P2RY2*, *ADORA1* and *ADORA3* expression in NOS and COPD patients and *P2RX2* and *ADORA2B* expression in COPD patients were also upregulated. No changes were found in *ADORA2A* expression in COPD patients. **(C)** No expression of *IL-13* or *IL-4* was found. *CCL18* expression was upregulated in NOS and downregulated in COPD patients. *Significantly different from NS (*p* < 0.05).

Similar patterns of gene expression of the ATP-degrading enzymes were found in the intercostal arteries compared to pulmonary tissue (Fig. 2A). *ENTPD1*, *ENTPD2* and *ENTPD3* expression was downregulated in NOS, while *ENTPD2* and *ENTPD3* expression was also reduced in COPD patients. As observed in the lung samples, *ENPP1* expression was upregulated in the intercostal arteries of both NOS and COPD patients. *NT5E* expression in the intercostal arteries was upregulated in NOS, as also seen in the lung tissue, but was reduced by up to one-fold in the intercostal arteries of COPD patients, which differed to that observed in the lungs. *ADA* and *DPP4* expression was upregulated in the intercostal arteries of both groups, which was opposite to that observed in the lungs.

#### Gene expression of P1 and P2 receptors

The expression patterns of the P1 and P2 receptors differed between the intercostal arteries and pulmonary tissue (Fig. 2B). In the intercostal arteries, *P2RX2* expression was upregulated in COPD patients and downregulated in NOS (*P2RX2* expression was downregulated in the lung tissues of both groups). *P2RX7* expression also differed between the tissues in both groups. Unlike in lung tissue, *P2RY2* expression was upregulated in the intercostal arteries of both groups. Whereas *ADORA1* expression was downregulated in the lung samples of COPD patients, it was upregulated by up to one-fold in the intercostal arteries of both groups, this being significant for the COPD patients when compared to NS. There were no major differences in the expression levels of *ADORA2A*, *ADORA2B* and *ADORA3* between the tissues.

#### Expression of the genes associated with inflammation

*IL-13* and *IL-4* expression was not amplified in the intercostal arteries, probably due to a lack of expression in these tissues (Fig. 2C). In NOS, *CCL18* expression differed between the intercostal arteries and lung tissue, being upregulated by up to one-fold in the intercostal arteries (Fig. 2C).

**Figure 3:**
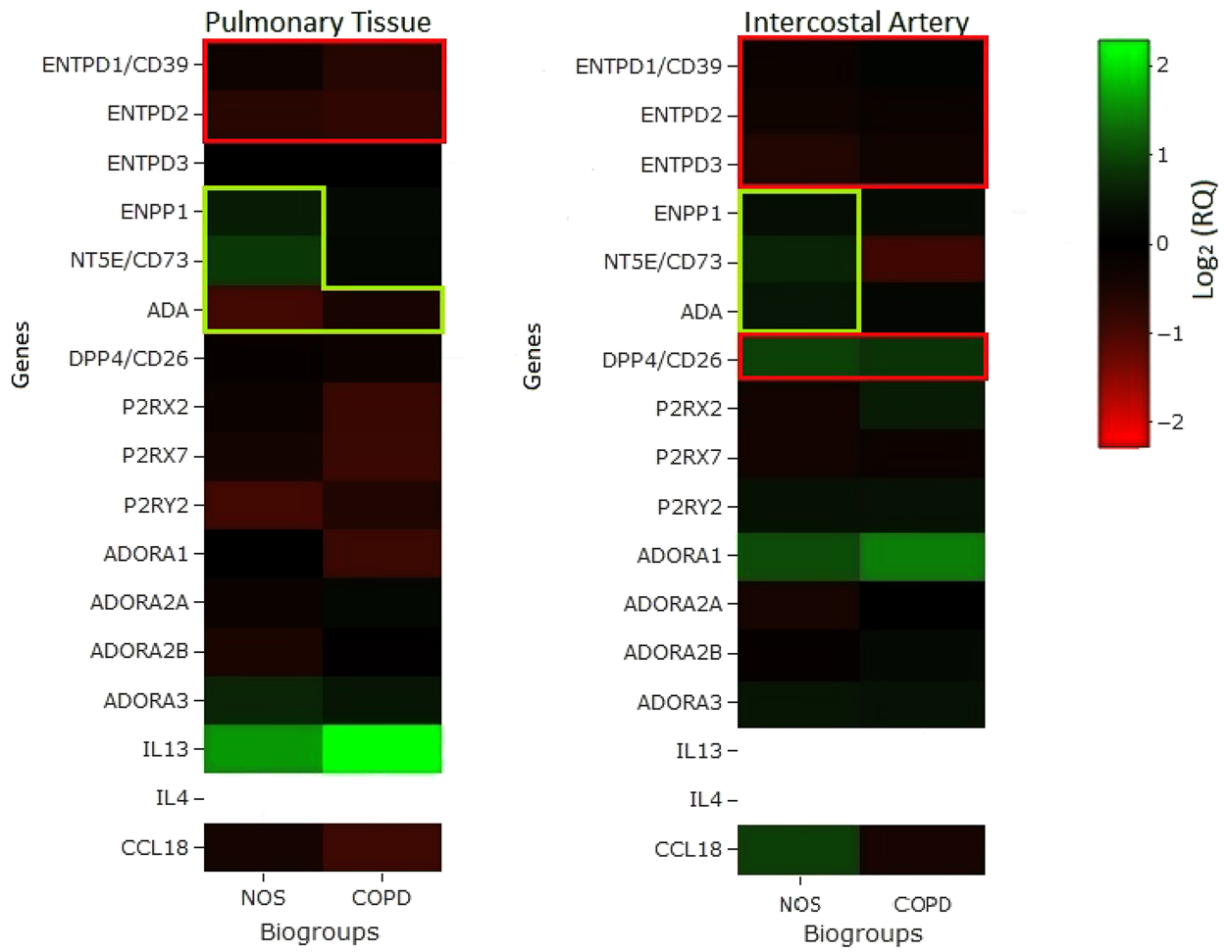
Schematic of the genes associated with purinergic signalling that were analysed. Heat map of the genes over- or underexpressed (log_2_ of RQ) in NOS and COPD patients compared to NS. Genes that promote or reduce inflammation are highlighted (red for promotion and green for reduction).

Figure 3 summarises the main and most interesting results in the format of a heat map. To facilitate the understanding of our findings regarding the enzymes associated with purinergic signalling and their relationship with inflammation, we have highlighted the genes that promote or reduce inflammation.

No significant differences were found in the expression of the purinergic pathway genes in lung or intercostal artery after adjusting for sex and diabetes. Furthermore, to analyse potential susceptibility according to gender, independently of tobacco consumption, we compared the gene expression specifically in the non-smoker group and found no significant differences (p > 0.05) in the expression of purinergic pathway genes.

### Comparison of gene expression levels in the lung and intercostal arteries between COPD patients and NOS

Changes in the gene expression levels in pulmonary tissue and intercostal arteries in COPD patients compared to NOS are shown in Table 1. All the ATP-degrading enzymes studied were downregulated in the lung, but upregulated or unaffected in the intercostal arteries of COPD patients compared to NOS. *NT5E* expression was downregulated in both tissues, while *ADA* expression was upregulated in the lung and downregulated in the intercostal arteries.

**Table 1:** Changes in gene expression in COPD patients compared to NOS.

	Pulmonary tissue	*p*-value	IC arteries	*p*-value
	Fold change (log_2_)		Fold change (log_2_)	
**Membrane purinergic degrading enzymes**				
*ENTPD1/CD39*	−0.366	0.458	0.332	0.608
*ENTPD2*	−0.129	0.861	0.116	0.902
*ENTPD3*	−0.009	0.987	0.253	0.627
*ENPP1*	−0.295	0.705	−0.032	0.906
*NT5E/CD73*	−0.671	0.386	−1.561	0.139
**Soluble purinergic degrading enzyme**				
*ADA*	0.472	0.330	−0.268	0.595
**ADA receptor**				
*DPP4/CD26*	−0.103	0.850	−0.137	0.745
**P2 receptors**				
*P2RX2*	−0.597	0.288	0.938	0.404
*P2RX7*	−0.472	0.461	0.093	0.884
*P2RY2*	0.374	0.366	0.010	0.983
**P1 receptors**				
*ADORA1*	−0.881	0.136	0.366	0.702
*ADORA2A*	0.429	0.129	0.471	0.487
*ADORA2B*	0.452	0.314	0.397	0.818
*ADORA3*	−0.211	0.739	−0.056	0.920
**Inflammatory genes**				
*IL-13*	0.684	0.356	—	—
*IL-4*	—	—	—	—
*CCL18*	−0.474	0.539	−1.365	0.121

In the lung, *P2RX2*, *P2RX7*, *ADORA1* and *ADORA3* expression levels were downregulated, while *P2RY2*, *ADORA2A* and *ADORA2B* expression was upregulated. In the intercostal arteries, all the genes for the P1 and P2 receptors, except *ADORA3*, were upregulated. Regarding the genes associated with inflammation, *IL-13* expression was upregulated in pulmonary tissue, but not expressed in the intercostal arteries. *IL-4* expression was not observed in the lung or intercostal arteries. *CCL18* expression was downregulated in both tissues.

## Discussion

The focus of our study was to characterise the expression patterns of the genes associated with purinergic signalling in the early phases of COPD. We also compared gene expression between pulmonary tissue and systemic arteries from the same patients to identify the mechanisms that initiate and perpetuate this disease and detect any potential molecules involved with the systemic vascular changes observed in COPD. This is the first study to explore the expression of the genes associated with purinergic signalling in the lung and systemic arteries of COPD patients using TaqMan low-density arrays.

**Figure 4:**
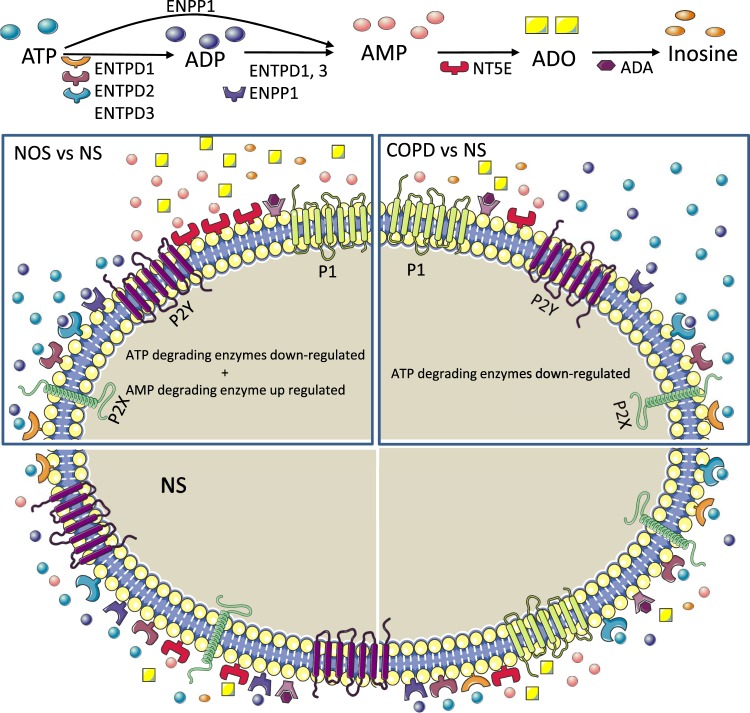
Graphic image representation of the possible mechanisms of lung inflammation in COPD involving purinergic signaling pathway. See text for details and further information (second paragraph of the discussion section). Pro-inflammatory molecules: ATP and derivates (dark and light blue and pink circles). Anti-inflammatory molecules: adenosine (yellow square). The figure was created using Servier Medical Art according to a Creative Commons Attribution 3.0 Unported License guidelines 3.0 (https://creativecommons.org/licenses/by/3.0/). Simplification and colour changes were made to the original cartoons.

Our results suggest that the downregulation of ATP-degrading and adenosine-producing enzymes in the lung and intercostal arteries could produce a pro-inflammatory state in COPD patients compared to NOS. Although ATP-degrading enzymes are also downregulated in both tissues in NOS, adenosine-generating enzymes are upregulated (Figs 1A and 2A), suggesting a compensatory mechanism that exists only in NOS. We found an imbalance between the enzymes regulating extracellular ATP and adenosine levels in the early stages of COPD that favoured ATP accumulation (acting as a pro-inflammatory molecule through the P2 receptors) and lowered adenosine levels (acting as anti-inflammatory molecules through the P1 receptors), possibly causing the overall pulmonary inflammation^18^. This imbalance could be overcome in NOS by an enhanced expression of *NT5E*, which encodes the main enzyme (CD73) regulating extracellular adenosine levels. Figure 4 summarizes all this information and represents the possible molecular mechanism in which changes in the expression of purinergic signalling genes favour the accumulation of extracellular ATP that may promote inflammation in COPD patients. By contrast, NOS balanced the levels of extracellular pro-and anti-inflammatory molecules through the increase of NT5E/CD73 expression.

Previous studies have shown that tobacco induces an accumulation of extracellular ATP in the human respiratory tract, leading to high ATP concentrations in COPD patients, even after smoking cessation^19^. By activating P2 receptors, ATP induces macrophages and neutrophils to secrete pro-inflammatory molecules and the mediators of tissue degradation, thus contributing to the chronic inflammation characteristic of COPD^15^. Extracellular adenosine levels are also increased in the lungs of patients with severe COPD. In accordance with this, previous studies have shown that the enzymatic activity of NT5E/CD73 is increased in the lung tissue of patients with severe COPD compared to smokers with mild obstruction^20^. In addition, COPD patients with acute exacerbations have decreased ADA enzymatic activity (inactivates adenosine), thus favouring its extracellular accumulation^21^. Adenosine has immunomodulatory functions; thus, its role may be important in COPD. In fact, adenosine receptors have been proposed as possible therapeutic targets in the treatment of COPD^22^. However, all the studies to date have focused on the advanced stages of the disease (GOLD stages III and IV) to assess the roles of ATP and adenosine in the pathophysiology of COPD. Given the dual role of adenosine depending on the situation, i.e., acting as an anti-inflammatory molecule in processes associated with acute lung diseases^23,24^ or as a pro-inflammatory agent with tissue remodeling functions in chronic lung diseases^25^, it is important to distinguish between the early and late stages of COPD when determining the role of adenosine in this disease. Furthermore, some adenosine receptors also act as anti- or pro-inflammatory molecules depending on the stages (acute or chronic) of lung injury during which they are activated^26^.

As seen in Fig. 3, NOS expressed more genes favouring a non-inflammatory state than COPD patients in both pulmonary and systemic tissues when compared to NS. The downregulated ectonucleotidases play a larger role in inflammation secondary to tobacco in the lung than in the arterial tissue of the same patients. This observation is in line with the previous studies of our group demonstrating an underexpression of ENTPDase1/CD39 in the lungs of COPD patients^27^. NT5E/CD73, which is expressed less in the lungs of COPD patients than NOS, is markedly decreased in systemic arteries. NT5E/CD73 has been reported to play an important anti-inflammatory role that is associated with anti-fibrotic activity and a reduced production of pro-inflammatory cytokines in the aortic artery, most likely by activating adenosine A2a receptors^28^. This would be interesting to investigate further in future studies assessing the role of CD73 in cardiovascular risk.

Classically, the inflammation leading to COPD has been described as a type I inflammation predominantly involving neutrophils. In line with this, the inflammatory parameters analysed in our patients, such as C-reactive protein (CRP) and leukocyte blood counts, were higher in COPD patients than NOS, even though these differences were not significant (Table 2). However, new evidence has emerged that type 2 or eosinophilic inflammation also plays a role in some COPD patients^29,30^. For this reason, we decided to analyse both *IL-13* and *IL-4* gene expression as they are involved in eosinophilic inflammation. Our results on *IL-13* expression in lung tissue provide a plausible explanation for why NOS show less inflammation than patients with moderate COPD (Fig. 1C). We analysed *IL-4* and *IL-13* expression to determine the type of inflammatory response elicited in our COPD patients. *IL-13* expression was significantly elevated in the pulmonary tissues of NOS and COPD patients, suggesting a Th2-derived inflammatory response (Fig. 1C). *IL-13* was not expressed in the intercostal arteries of NOS or COPD patients, suggesting that this type of inflammation is not relevant in the initial systemic vascular changes in smokers and patients with moderate COPD. As for *IL-4*, we could not assess any amplification of this gene in the lung or intercostal arteries (Figs 1C and 2C). We were not able to determine if this was because there was no expression or whether this was due to methodological issues. *CCL18*, a chemokine involved in vascular changes^31^, showed reduced expression in the lungs and systemic arteries of COPD patients. Other studies have shown a similar gene expression pattern of CCL18 in COPD patients and smokers^31^.

**Table 2:** Clinical parameters and lung function measurements of the subjects.

Parameters	NS (n = 16)	NOS (n = 17)	COPD (n = 21) GOLD I/II/III: 14/5/2	*p*-value
Gender, female/male	11/5	0/17	2/19	<0.0001
Age, years	61.7 ± 12.1	61.6 ± 11.1	62.8 ± 8.5	0.928
BMI, kg/m²	27 ± 4.2	27.8 ± 4.4	25.7 ± 4.6	0.342
Smoking history, pack/years	—	38.9 ± 4.4	48 ± 3.5	0.113
Current smokers, n (%)	—	9 (52.9)	5 (23.8)	0.067
HTA, n (%)	4 (25)	8 (47.1)	9 (42.9)	0.384
DLP, n (%)	8 (50)	9 (52.9)	11(52.4)	0.984
DM, n (%)	0 (0)	8 (47)	1 (4.8)	<0.0001
FVC, % predicted	113.8 ± 21	99.4 ± 16.6	89.8 ± 14.1	<0.001
FEV_1_, % predicted	110.1 ± 20.3	95.4 ± 14.5	67 ± 15.7	<0.001
FEV_1_/FVC, %	77.4 ± 5.6	76 ± 5.1	56.2 ± 11.4	<0.001
D_LCO_, % predicted	93.6 ± 18	87.2 ± 17.3	69.5 ± 14.7	<0.001
Fibrinogen, g/L	3.1 ± 1.3	3.2 ± 1	3.2 ± 0.9	0.937
Leukocytes, cells/mm³	6.8 ± 1.5	8.3 ± 1.9	8.7 ± 1.8	<0.05
Eosinophils, cells/mm^3^	134.4 ± 54	84.1 ± 42.7	116.2 ± 73	0.056
CRP, mg/L	3.6 ± 5	6.8 ± 10.2	8 ± 11.2	0.463
Cholesterol, mmol/L	4.7 ± 1	4.4 ± 0.8	4.5 ± 0.9	0.582

Data are presented as mean ± SD. BMI, body mass index; HTA, arterial hypertension; DLP, dyslipidaemia; DM, diabetes mellitus; FVC, forced vital capacity; FEV_1_, forced expiratory volume in first second; D_LCO_, diffusing capacity for carbon monoxide; CRP, C-reactive protein.

There were differences in purinergic signalling between patients with moderate COPD and NOS. However, other studies have found some similarities in the expression profiles of other genes between patients with moderate COPD and NOS, which have not been observed in patients with severe COPD^32^. For this reason, we believe that this study should be complemented with future studies investigating pulmonary levels of ATP and adenosine and the genetic expression of purinergic signalling enzymes in patients with severe COPD (GOLD stages III and IV) too.

This study had several strengths and limitations. Performing a genetic analysis on patients in the early stages of COPD enabled us to check for disease-initiating mechanisms that are more difficult to detect at later stages of the disease. Nevertheless, it has to be pointed out that this is an exploratory study to generate new hypotheses about the pathophysiological changes that occur in the first steps of COPD. Almost none of the *p*-values of the results comparing gene expression levels between the groups were statistically significant, probably because of the exploratory nature of the study and/or the small number of patients included. The study population had primary treatable lung cancer and, therefore, lung cancer could have been a possible introduced bias. However, we assumed that any bias introduced would have been the same for all the subjects. Moreover, this was the only way of obtaining fresh tissue samples from the patients along with clinical and functional data.

In summary, this preliminary study suggests that the expression patterns of different extracellular ATP-degrading enzymes are altered in a manner that promotes inflammation in both NOS and patients with early COPD, with a compensatory mechanism possibly occurring only in NOS. *ENTPD1*, *ENTPD*2 and *NT5E* might be relevant in the pathophysiology of COPD. Future studies are needed to confirm this hypothesis.

## Materials and Methods

### Subjects

Fifty-four patients who underwent a lobectomy or pneumonectomy of a solitary pulmonary nodule at Bellvitge University Hospital were included in this study. Demographic, clinical and pre-operative pulmonary function assessment (spirometry, lung volumes and carbon monoxide diffusing capacity) data were collected for all the subjects before surgery. None of the patients presented severe systemic comorbidities, atelectasis or obstructive pneumonitis. Moreover, they had not received chemotherapy or radiotherapy prior to surgery. According to their previous smoking history and the results of the pulmonary function tests, subjects were classified as follows: 16 never smokers (NS), 17 non-obstructed smokers (NOS) and 21 stable COPD patients. COPD was diagnosed based on current GOLD guidelines^33^. In the COPD group, most of the patients had early stages of disease (14 GOLD I and five GOLD II). All the participants signed an informed consent in accordance with the principles outlined in the Declaration of Helsinki. The study was approved by the local ethics committee (CEIC, ref. PR330/15). General characteristics and lung function measurements of the three groups are summarised in Table 2.

### Sample collection and processing

The samples used in this study were obtained and processed as previously described^9^. They have been used in previous studies published by our research group^9,27,31^.

### RNA processing

Total RNA was isolated and purified as previously described^27,31^.

### TaqMan low-density arrays

Gene expression analysis was performed by real-time PCR using Custom TaqMan low-density arrays (TLDAs; Applied Biosystems, Foster City, CA, USA). The 17 genes analysed in this study are listed in Table 3, classified by their functional groups. One µg of total RNA was retrotranscribed into cDNA using the High-Capacity cDNA Reverse Transcription Kit (Applied Biosystems). Before performing PCR, cDNA was pre-amplified using Custom TaqMan PreAmp Pools, following the manufacturer’s protocol (Applied Biosystems). PCR reactions were prepared with the TaqMan Gene Expression Master Mix, following the manufacturer’s protocol (Applied Biosystems), and samples were run in triplicate on an ABI Prism 7900HT Real-Time PCR System (Applied Biosystems). Data were collected using SDS Software version 2.4 (Applied Biosystems) and used for subsequent analysis.

**Table 3:** List of genes analysed in the study.

Gene symbol	Gene name	Assay ID
**Membrane purinergic degrading enzymes**		
*ENTPD1/CD39*	Ectonucleoside triphosphate diphosphohydrolase 1	Hs00969559_m1
*ENTPD2*	Ectonucleoside triphosphate diphosphohydrolase 2	Hs00993193_g1
*ENTPD3*	Ectonucleoside triphosphate diphosphohydrolase 3	Hs00154325_m1
*ENPP1*	Ectonucleotide pyrophosphatase/phosphodiesterase 1	Hs01054040_m1
*NT5E/CD73*	5′-nucleotidase ecto	Hs04234687_m1
**Soluble purinergic degrading enzyme**		
*ADA*	Adenosine deaminase	Hs01113256_g1
**ADA receptor**		
*DPP4/CD26*	Dipeptidyl peptidase 4	Hs00897386_m1
**P2 receptors**		
*P2RX2*	Purinergic receptor P2X2	Hs04176268_g1
*P2RX7*	Purinergic receptor P2X7	Hs00951607_m1
*P2RY2*	Purinergic receptor P2Y2	Hs04176264_s1
**P1 receptors**		
*ADORA1*	Adenosine A1 receptor	Hs00379752_m1
*ADORA2A*	Adenosine A2a receptor	Hs00169123_m1
*ADORA2B*	Adenosine A2b receptor	Hs00386497_m1
*ADORA3*	Adenosine A3 receptor	Hs04194761_s1
**Inflammatory genes**		
*IL-13*	Interleukin 13	Hs01124272_g1
*IL-4*	Interleukin 4	Hs00929862_m1
*CCL18*	C-C motif chemokine ligand 18/PARC	Hs00268113_m1

### Gene expression analysis

Data analysis was performed using the Relative Quantification application module on the Thermo Fisher Cloud online software (https://www.thermofisher.com/es/es/home/cloud.html). Relative quantification (RQ) was based on the comparative cycle threshold (Ct) method using *GAPDH* (Hs99999905_m1) as an endogenous control. For differential expression analysis, a limma-modified t-test^34^ was used to calculate the ΔΔCt value [ΔΔCt = mean ΔCt value (target samples) - mean ΔCt value (control samples)]. RQ was calculated from these ΔΔCt values (RQ = 2^−ΔΔCt^) and used for fold change calculations. Results are expressed as fold changes in logarithms to base 2 (log_2_) of the RQ values.

Heat maps were created with the plotly and ggplot2 R packages version 3.5 (R Foundation for Statistical Computing, Vienna, Austria; https://www.R-project.org/).

### Statistical analysis

Continuous variables were compared by Student’s t-test and expressed as mean ± standard deviation (SD). Qualitative variables were compared with the chi-square test. Comparisons between the groups were evaluated by one-way analysis of variance (ANOVA) and an overall *p*-value was calculated. Adjusted analyses were performed using unbalanced demographic variables (gender and diabetes). Statistical analysis was performed using IBM SPSS version 19.0 (IBM Corp., Armonk, NY, USA). A *p*-value less than 0.05 indicated statistical significance.
